# Aspirin reduces the mortality risk of sepsis-associated acute kidney injury: an observational study using the MIMIC IV database

**DOI:** 10.3389/fphar.2023.1186384

**Published:** 2023-07-25

**Authors:** Sining Chen, Shishi Li, Chaoying Kuang, Yuzhen Zhong, Zhiqian Yang, Yan Yang, Fanna Liu

**Affiliations:** Nephrology Department, The First Affiliated Hospital of Jinan University, Jinan University, Guangzhou, China

**Keywords:** sepsis-associated acute kidney injury, sepsis, aspirin, critically ill, mimic iv, mortality

## Abstract

**Introduction:** Sepsis-associated acute kidney injury (SA-AKI) is a complication of sepsis and is characterized by high mortality. Aspirin affects cyclooxygenases which play a significant role in inflammation, hemostasis, and immunological regulation. Sepsis is an uncontrolled inflammatory and procoagulant response to a pathogen, but aspirin can inhibit platelet function to attenuate the inflammatory response, thus improving outcomes. Several studies have generated contradictory evidence regarding the effect of aspirin on patients with sepsis-associated acute kidney injury (SA-AKI). We conducted an analysis of the MIMIC IV database to investigate the correlation between aspirin utilization and the outcomes of patients with SA-AKI, as well as to determine the most effective dosage for aspirin therapy.

**Materials and methods:** SA-AKI patients’ clinical data were extracted from MIMIC-IV2.1. Propensity score matching was applied to balance the baseline characteristics between the aspirin group and the non-user group. Subsequently, the relationship between aspirin and patient death was analyzed by Kaplan-Meier method and Cox proportional hazard regression models.

**Results:** 12,091 patients with SA-AKI were extracted from the MIMIC IV database. In the propensity score-matched sample of 7,694 individuals, lower 90-day mortality risks were observed in the aspirin group compared to the non-users group (adjusted HR: 0.722; 95%CI: 0.666, 0.783) by multivariable cox proportional hazards analysis. In addition, the Kaplan-Meier survival curves indicated a superior 90-day survival rate for aspirin users compared to non-users (the log-rank test *p*-value was 0.001). And the median survival time of patients receiving aspirin treatment was significantly longer than those not receiving (46.47 days vs. 24.26 days). In the aspirin group, the average ICU stay length was shorter than non-users group. (5.19 days vs. 5.58 days, *p* = 0.006). There was no significant association between aspirin and an increased risk of gastrointestinal hemorrhage (*p* = 0.144).

**Conclusion:** Aspirin might reduce the average ICU stay duration and the 30-day or 90-day mortality risks of SA-AKI patients. No statistically significant difference in the risk of gastrointestinal hemorrhage was found between the aspirin group and the control group.

## 1 Introduction

Sepsis is an uncontrolled host response to infection, which may result in severe organ failure if left untreated. The diagnostic criteria were updated in The Third International Consensus Definitions Task Force: in ICU, patients who are suspected of infection and having a Sepsis-related Organ Failure Assessment (SOFA) score ≥2 (Seymour et al., 2016). Overall, 40%–50% of septic patients exhibit acute kidney injury, namely, sepsis-associated acute kidney injury (SA-AKI), which is one of the most frequent and serious complications of sepsis (Gómez and Kellum, 2016). SA-AKI is characterized by high mortality and poor outcomes (He et al., 2022). The mainstay of SA-AKI treatment comprises supportive measures, such as renal replacement therapy (RRT), maintaining balance in fluids, acid-base and electrolytes homeostasis, nutritional supplementation, and sustaining hemodynamic stability (Ricci et al., 2011). Furthermore, previous research found that microvascular dysfunction can activate platelets to promote the progression of SA-AKI (Peerapornratana et al., 2019).

Aspirin is a widely used drug that has antipyretic, analgesic, and anti-inflammatory properties (Montinari et al., 2019) due to its effects on cyclooxygenases, which play a significant role in inflammation, hemostasis, and immunological regulation (Menter and Bresalier, 2022). Further research has revealed additional applications for aspirin, including antiplatelet aggregation to reduce the risk of recurrence of myocardial infarction in cardiovascular disease patients (Patrono et al., 2011)and prevention of colorectal cancer (Drew et al., 2016).

Sepsis is thought to be a dysregulated inflammatory and procoagulant response to pathogens, with platelets interacting with endothelial cells and regulating the immune system. Aspirin can inhibit platelet function and attenuate the inflammatory response, thereby improving outcomes (Akinosoglou and Alexopoulos, 2014). Several animal studies have reported aspirin as a promising treatment option for sepsis (Hinshaw et al., 1967; Halushka et al., 1981) and SA-AKI (Silva et al., 2021). However, it contradicts the popular view that using non-steroidal anti-inflammatory drugs for extended periods of time may harm the kidney, leading to chronic interstitial nephritis or renal papillary necrosis (Lafrance and Miller, 2009). Aspirin was given to rats (120–230 mg/kg/day) for 40–83 weeks, which resulted in renal papillary necrosis and a decline in the urine concentration capacity (Burrell et al., 1991). Sossdorf et al. have indicated that the administration of aspirin resulted in a reduction in mortality among ICU patients with sepsis (Sossdorf et al., 2013). However, Al Harbi et al. have reported that aspirin was linked to a higher risk of severe sepsis acquired in the ICU and did not reduce ICU mortality (Al Harbi et al., 2016). Eisen et al. have discovered that aspirin was linked to a decreased risk of mortality in sepsis patients but was also associated with an increased risk of renal injury (Eisen et al., 2012). These studies have generated contradictory evidence regarding the effect of aspirin on patients with sepsis-associated acute kidney injury (SA-AKI).

Although there are several studies reported that anti-platelet drugs can decrease risk of mortality in sepsis patients (Ouyang et al., 2019) and patients with AKI(Jansen et al., 2018), a limited number of clinical studies have investigated the association between aspirin and SA-AKI patients. And aspirin is a special antiplatelet drug because different dosage of aspirin has different function (Patrono and Baigent, 2019). Low-dose aspirin (75–100 mg/d) has anti-platelet effects and high-dose aspirin (>300 mg/d) has anti-inflammatory effects besides anti-platelet effects. Previous studies did not take into account the optimal dose of aspirin in sepsis patients. Previous studies focused on the influence of low-dose aspirin in sepsis patients (Eisen et al., 2012; Sossdorf et al., 2013). Hence, we conducted an analysis of the MIMIC IV database to investigate the correlation between aspirin utilization and the outcomes of patients with SA-AKI, as well as to determine the most effective dosage for aspirin therapy.

## 2 Materials and methods

### 2.1 Data source: MIMIC-IV

The Medical Information Mart for Intensive Care IV (MIMIC-IV) database (https://physionet.org/content/mimiciv/2.1/) is a single-center and open-access database containing 730,141 ICU admissions from the Beth Israel Deaconess Medical Center from 2008 to 2019, located in the United States (Johnson et al., 2022). Shishi Li, one of the authors, collected clinical data from the MIMIC database (certification number: 42257067), including patient demographic information, laboratory findings, and medication. The use of the database was approved by the Massachusetts Institute of Technology and Beth Israel Deaconess Medical Center’s Institutional Review Boards. This project complied with the Helsinki Declaration and approval from the ethics committee was not required due to participant anonymity and data standardization in this database.

## 3 Inclusion and exclusion criteria

Sepsis patients who met the diagnostic criteria, Sepsis-3 guidelines (Singer et al., 2016), were eligible for inclusion in the study. Among these sepsis patients, individuals with AKI based on the Kidney Disease Improving Global Outcome (KDIGO) criteria (Khwaja, 2012) were selected as research subjects. Both SCr and urine volume were used to categorize the stages of AKI. The exclusion criteria were: 1) ICU stay duration < 48 h (discharged or death); 2) Patients aged < 18 years. The SA-AKI patients who received aspirin in the hospital were compared to those who did not.

### 3.1 Data collection

Structured query language (SQL) was used to obtain patient information from MIMIC-IV2.1 in Navigate Premium (version 16). The code was obtained from https://github.com/MIT-LCP/mimic-iv/concepts_postgres. Patient demographics were then obtained, including age, gender, and ethnicity. Furthermore, vital signs were measured as soon as patients were hospitalized, including systolic blood pressure, diastolic blood pressure, heart rate, and oxygen saturation (SpO2). The following SA-AKI comorbidities were recorded: hypertension, diabetes mellitus, and cardiac surgery. Subsequently, the laboratory indexes within the first day of ICU admission were extracted, including hemoglobin, white blood cells, platelets, glucose, prothrombin time, and urine output. SOFA scores and GCS scores were calculated at ICU admission. The following treatment information was noted: use of mechanical breathing, vasopressors, and renal replacement therapy (RRT).

### 3.2 Primary outcome and secondary outcomes

The primary outcome of this study was the 90-day mortality. The secondary outcomes included 30-day mortality, ICU stay duration, and gastrointestinal hemorrhage.

### 3.3 Statistical analysis

All variables in our study had fewer than 5% missing values. The missing values were replaced using single imputation (Supplementary Table S1). SA-AKI patients treated with aspirin during hospitalization were defined as the experimental group, whereas those who did not receive aspirin constituted the control group. The Mann–Whitney *U* test was used for non-normally distributed continuous variables and were expressed by the median and interquartile range. The categorical variables of the two groups were expressed by numbers with proportions and were compared using the chi-square test. All data were analyzed using SPSS Statistics (version 23.0). Statistical significance was defined as less than or equal to 0.05.

Propensity score matching (caliper value: 0.02) was used to narrow the gap of baseline characteristics between the two groups.

The Kaplan-Meier curves indicated the occurrence of 30-day and 90-day deaths in SA-AKI patients and the median survival time. The Cox proportional-hazards model was used to estimate the relationships between SA-AKI patients treated or untreated with aspirin, the 90-day mortality and the 30-day mortality. Furthermore, SA-AKI patients were divided into different subgroups based on age, gender, race, AKI stage, co-morbidity, SOFA scores, GCS score, renal replacement therapy (RRT), and vasoactive drug, which were analyzed for HR and 95% CI separately.

## 4 Results

### 4.1 Patient characteristics

**FIGURE1:**
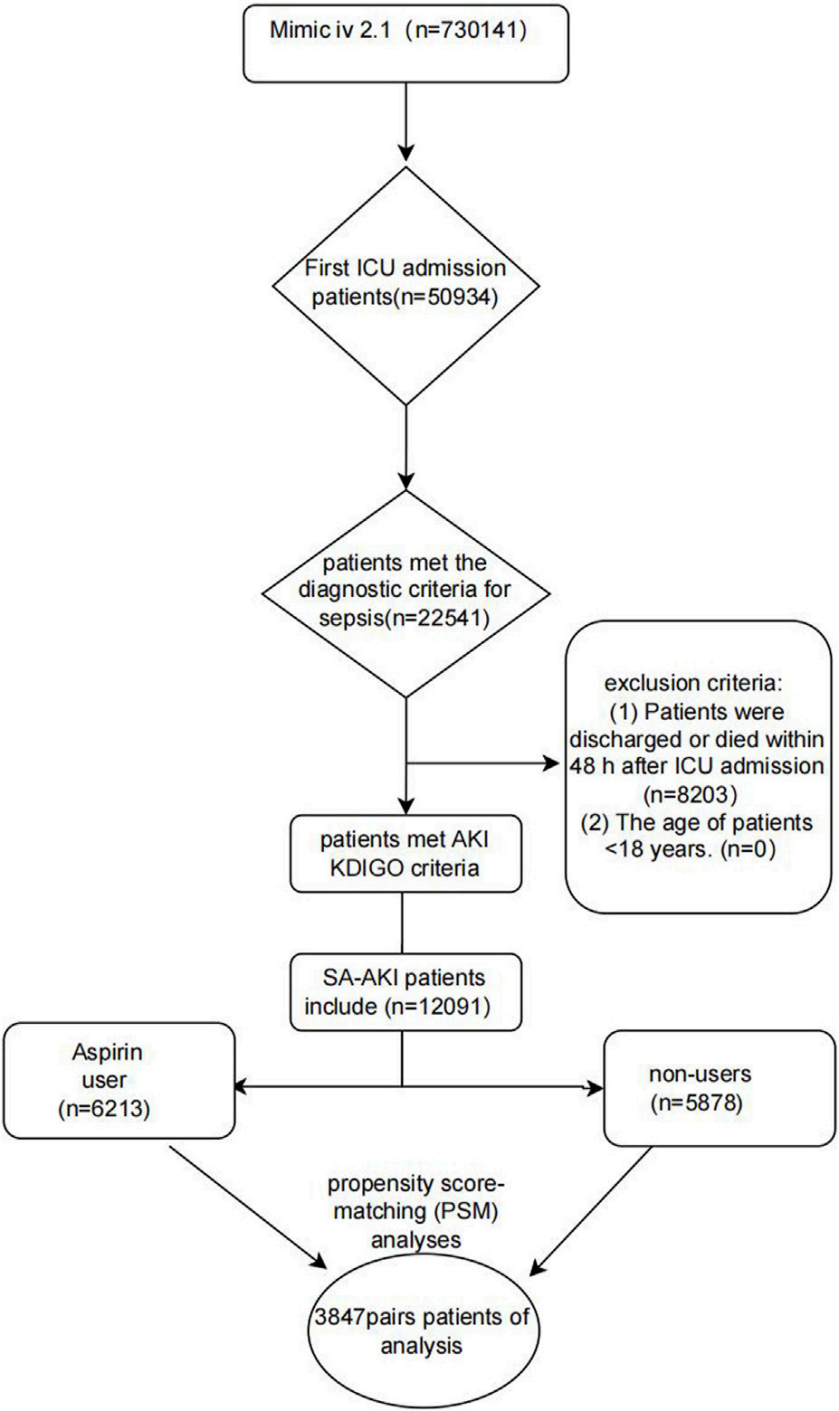
Flow diagram of the research.

Figure 1 presents the patient selection procedure in the current research. In total, 12,091 SA-AKI patients met the inclusion criteria. The detail clinical information of 12,091 patients with SA-AKI was shown in Supplementary Table S2 including the start time, end time, and dose of aspirin treatment (the non-user aspirin group is blank). The most common dose of aspirin is 81 mg/d (4,279 patients). In our study, there are 1,596 patients using high-dose aspirin (>300 mg/d). Our study is a retrospective study whose result is easily affected by confounding factors. Propensity score matching (PSM), a common method to minimize the effects of confounder, is to use propensity score to match individuals with the same or similar background characteristics from the control group for individuals in the experimental group. 12,091 patients with SA-AKI were extracted from the MIMIC IV database. 7,694 individuals were matched by PSM. After PSM, the interference of confounder was minimized and the main factor was administration of aspirin. Table 1 displays the clinical information of SA-AKI patients of SA-AKI patients in the two groups before or after Propensity score matching (PSM). During hospitalization, 6,213 SA-AKI patients were treated with aspirin, while 5,878 SA-AKI patients did not receive aspirin. 2,167 SA-AKI patients were classified as AKI stage 1 patients, 5,614 were stage 2, and 4,310 were stage 3. Moreover, 3,954 (32.7%) SA-AKI patients had diabetes mellitus, 3,568 (29.5%) SA-AKI patients had hypertension, and 5,122 (42.4%) SA-AKI patients had cardiac surgery. Compared to participants who did not receive aspirin, patients on aspirin showed a higher prevalence of hypertension (39.4%), diabetes mellitus (33.2%), and cardiac surgery (57.1%). In addition, the average age of the aspirin group was higher than the non-users group. The influence of confounding variables was reduced and the comparison between the experimental and control groups was adjusted by applying the PSM method with a caliper value of 0.02. Ultimately, 3,847 pairs of SA-AKI patients were matched. After PSM, the major clinical information between the aspirin users and non-users was compared (Table 1).

**TABLE 1:** Clinical information of SA-AKI patients before PSM and after PSM.

	Before PSM				After PSM			
	All patients (n = 12,091)	Non-users (n = 5,878)	Aspirin users (n = 6,213)	p	All patients (n = 7,694)	Non-users (n = 3,847)	Aspirin users (n = 3,847)	p
Age (years)	68.59 (57.16.79.38)	63.53 (51.70.76.31)	72.19 (62.79.81.00)	<0.001	69.25 (58.81.79.74)	68.81 (58.03.80.24)	69.56 (59.77.79.28)	0.273
Gender [male, n (%)]	6,939 (57.4)	3,208 (54.6)	3,731 (60.1)	<0.001	4,331 (56.3)	2,157 (56.1)	2,174 (56.5)	0.696
Ethnicity, n (%)								
White	7,975 (66.0)	3,723 (63.3)	4,252 (68.4)	<0.001	5,040 (65.5)	2,518 (65.5)	2,522 (65.6)	0.292
Yellow	295 (2.4)	157 (2.7)	138 (2.2)		184 (2.4)	104 (2.7)	80 (2.1)	
Black	975 (8.1)	514 (8.7)	461 (7.4)		674 (8.8)	327 (8.5)	347 (9.0)	
Other	2,846 (23.5)	1,484 (25.2)	1,362 (21.9)		1796 (23.3)	898 (23.3)	898 (23.3)	
Heart rate (bpm)	85.93 (75.92.98.27)	89.73 (77.71,102.72)	83.12 (74.67.93.35)	<0.001	86.13 (75.68.98.00)	86.50 (75.32.98.64)	85.85 (76.13.97.19)	0.735
Systolic pressure (mmHg)	112.83 (104.67,123.88)	113.31 (104.34,125.71)	112.47 (104.95,122.34)	0.006	113.34 (104.70,125.62)	113.40 (104.40,125.91)	113.24 (104.97,125.09)	0.829
Diastolic pressure (mmHg)	60.04 (54.10.66.90)	61.70 (55.56.68.96)	58.47 (52.83.64.82)	<0.001	60.43 (54.48.67.18)	60.56 (54.62.67.28)	60.33 (54.33.67.12)	0.493
SpO2 (%)	97.40 (95.88.98.70)	97.24 (95.68.98.68)	97.54 (96.04.98.73)	<0.001	97.31 (95.78.98.67)	97.28 (95.75.98.68)	97.34 (95.83.98.66)	0.627
**Comorbidities, n (%)**								
Diabetes mellitus	3,954 (32.7)	1,508 (25.7)	2,446 (39.4)	<0.001	2,535 (32.9)	1,243 (32.3)	1,292 (33.6)	0.235
Hypertension	3,568 (29.5)	1,504 (25.6)	2064 (33.2)	<0.001	2,292 (29.8)	1,140 (29.6)	1,152 (29.9)	0.765
Cardiac surgery	5,122 (42.4)	1,576 (26.8)	3,546 (57.1)	<0.001	2,792 (36.3)	1,378 (35.8)	1,414 (36.8)	0.393
**Therapy, n (%)**								
RRT	1,565 (12.9)	779 (13.3)	786 (12.7)	0.324	998 (13.0)	494 (12.8)	504 (13.1)	0.734
Vasoactive drug	7,466 (61.7)	3,223 (54.8)	4,243 (68.3)	<0.001	4,523 (58.8)	2,244 (58.3)	2,279 (59.2)	0.418
Mechanical ventilation	11,633 (96.2)	5,574 (94.8)	6,059 (97.5)	<0.001	7,416 (96.4)	3,700 (96.2)	3,716 (96.6)	0.328
**laboratory index**								
Urine output (ml)	1,425.00 (850.00,2135.00)	1,365.00 (788.75,2105.35)	1,470.00 (903.00,2150.00)	<0.001	1,373.50 (820.00,2090.50)	1,353.00 (799.00,2055.00)	1,400.00 (841.00,2125.00)	0.068
Hemoglobin (g/dL)	9.70 (8.20.11.40)	9.90 (8.30.11.60)	9.50 (8.20.11.10)	<0.001	9.90 (8.40.11.50)	9.90 (8.30.11.60)	9.80 (8.40.11.40)	0.695
White blood cell (× 109/L)	10.00 (7.10.13.50)	10.00 (6.70.13.80)	10.00 (7.40.13.20)	0.089	10.10 (7.10.13.60)	10.00 (6.90.13.70)	10.20 (7.40.13.60)	0.007
Platelets (× 109/L)	161.00 (109.00,225.00)	163.00 (103.00,232.00)	159.00 (114.00,218.00)	0.430	169.00 (114.00,232.00)	169.00 (111.00,233.00)	168.00 (117.00,231.00)	0.234
Glucose (mg/dL)	114.00 (95.00,137.00)	112.00 (93.00,136.00)	116.00 (98.00,139.00)	<0.001	115.00 (96.00,140.00)	115.00 (95.00,139.00)	116.00 (97.00,140.00)	0.196
Prothrombin time s)	13.70 (12.30.15.40)	14.00 (12.30.16.30)	13.50 (12.20.15.13)	<0.001	13.70 (12.20.15.50)	13.80 (12.20.15.80)	13.60 (12.30.15.20)	0.017
GCS score	12.00 (8.00.14.00)	11.0 (7.00.14.00)	13.00 (8.00.14.00)	<0.001	12.00 (7.00.14.00)	12.00 (7.00.14.00)	12.00 (7.00.14.00)	0.777
SOFA score	7.00 (5.00.10.00)	7.00 (5.00.11.00)	7.00 (5.00.9.00)	<0.001	7.00 (5.00.10.00)	7.00 (4.00.10.0	7.00 (5.00.10.0	0.078
AKI stage; n (%)								
1	2,167 (17.9)	1,018 (17.3)	1,149 (18.5)	<0.001	1,272 (16.5)	661 (17.2)	611 (15.9)	0.195
2	5,614 (46.4)	2,582 (43.9)	3,032 (48.8)		3,600 (46.8)	1767 (45.9)	1833 (47.6)	
3	4,310 (35.6)	2,278 (38.8)	2032 (32.7)		2,822 (36.7)	1,419 (36.9)	1,403 (36.5)	

Note: The Chi-square test was used to compare categorical variables, and the Wilcoxon rank-sum test was used for continuous variables.

### 4.2 Association between aspirin and mortality outcomes

**FIGURE 2:**
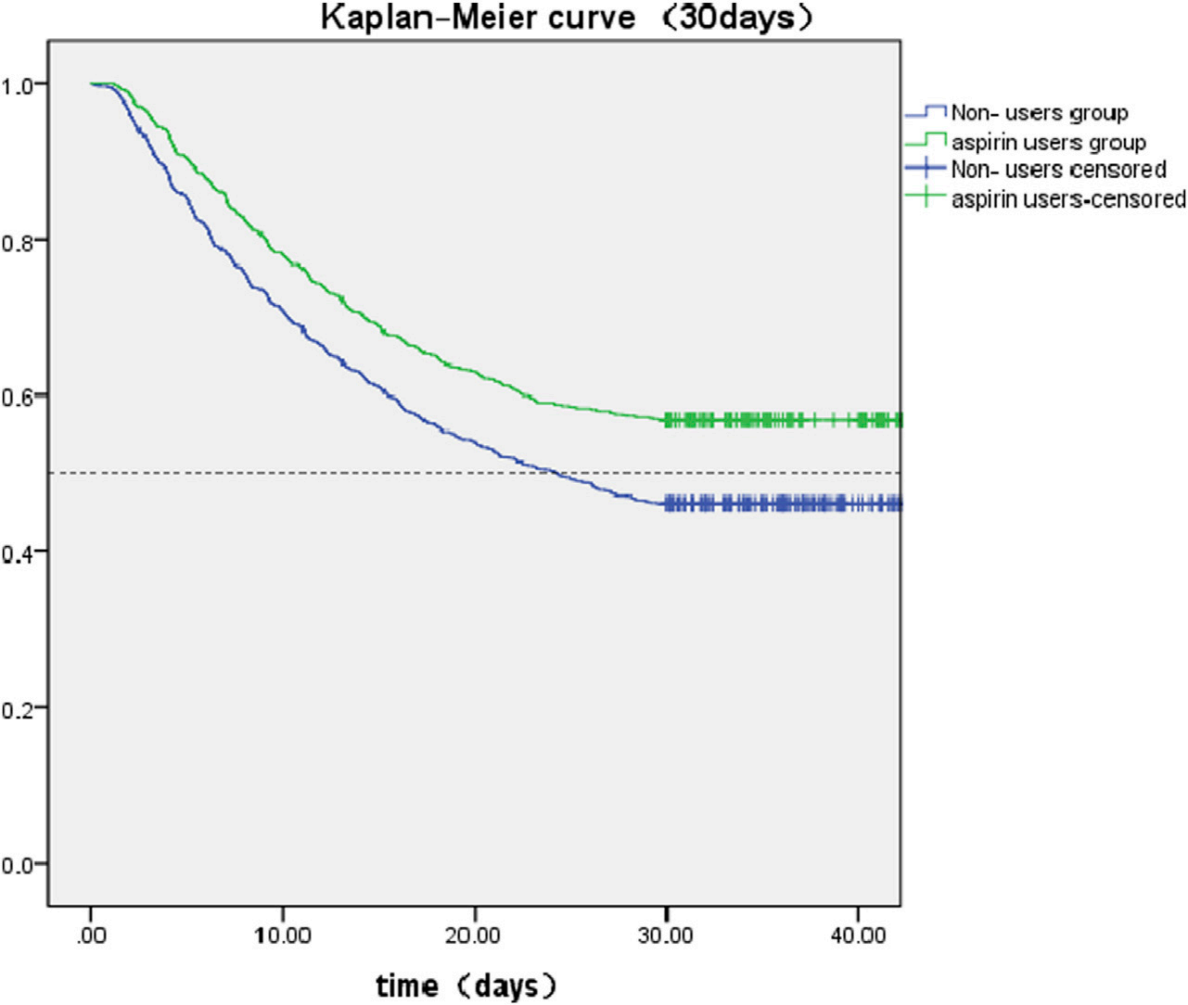
Kaplan-Meier survival curves between two groups indicated the 30-day mortality risk for the SA-AKI patients. Non-aspirin users are represented by blue lines and aspirin users are represented by green lines.

**FIGURE 3:**
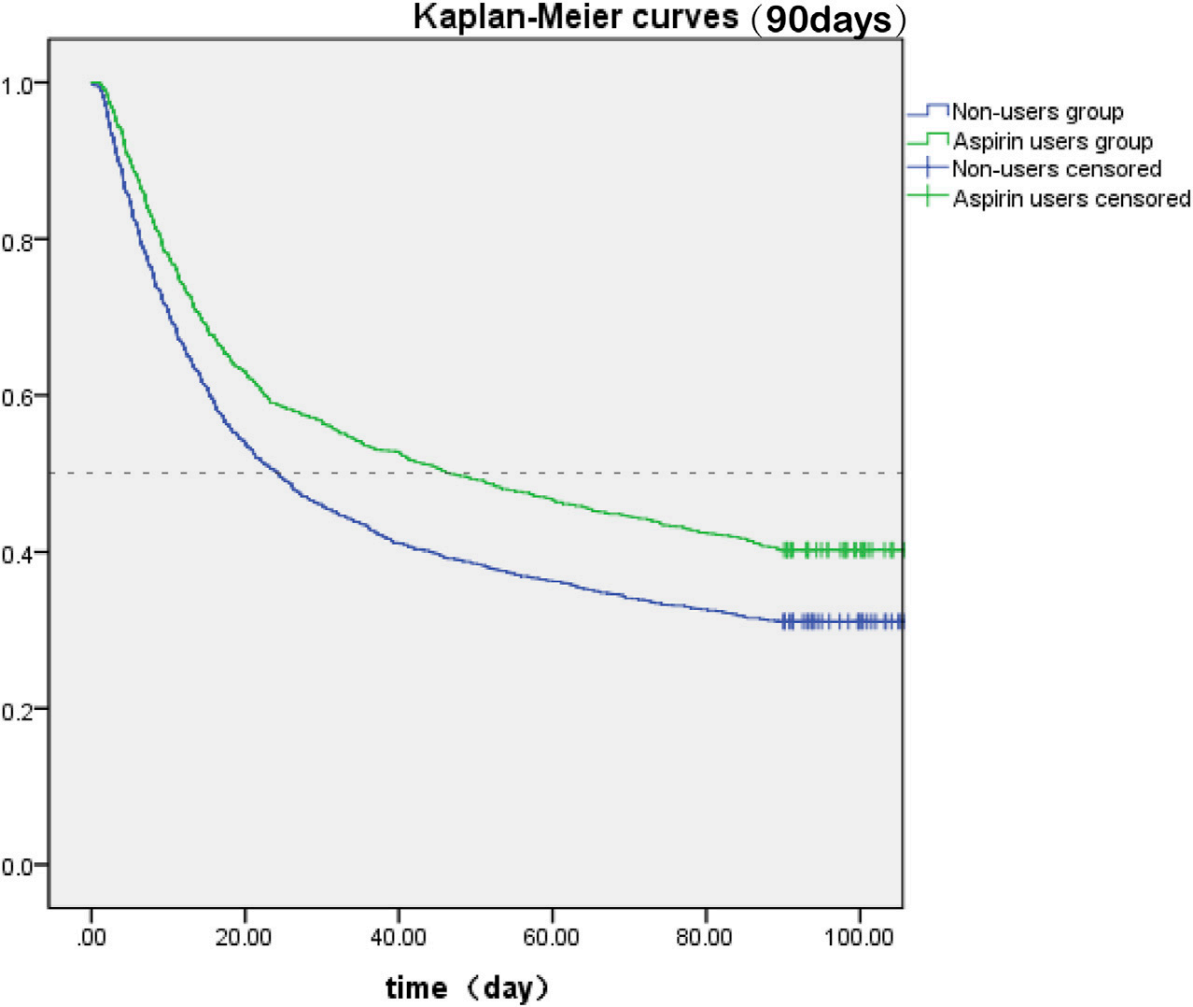
Kaplan-Meier survival curves between two groups indicated the 90-day mortality risk for the SA-AKI patients. Non-aspirin users are represented by blue lines and aspirin users are represented by green lines.

The SA-AKI patients were classified into two groups depending on whether they received aspirin during hospitalization. Lower 30- and 90-day mortality risks were observed in the aspirin group compared to the non-users group [HR: 0.727; 95% confidence interval (CI): 0.663–0.798 and HR: 0.759; 95% confidence interval (CI): 0.700–0.822] by univariate cox hazard analysis (Table 2). The Kaplan–Meier curve for 30-day survival was shown in Figure 2 and for 90-day survival was shown in Figure 3. According to Kaplan–Meier survival analysis, the aspirin users group had a significantly higher 30-day survival rate and 90-day survival rate than the non-users group (the log-rank test: *p*-value < 0.001). And the median survival time of patients receiving aspirin treatment was significantly longer than those not receiving (46.47 days vs. 24.26 days) (table 2). We analyzed the relationship between aspirin and the mortality of SA-AKI patients by cox regression analysis along with other data which may influence survival rate. Lower 30-day mortality risk and Lower 90-day mortality risk were observed in the aspirin group compared to the non-users group (adjusted HR = 0.689, 95%CI: 0.627, 0.757; adjusted HR = 0.722; 95%CI: 0.666, 0.783) by multivariable cox proportional hazards analysis (Table2).

**TABLE 2:** Survival outcomes of the aspirin group and the non-users group.

	Group	No.events/No. All patients	Median survival time (95%CI)	Log rank test value	Hazard ratio (95%CI)	Adjusted hazard Ratio (95%CI)
**30-day mortality**	Non-users	1,087/3,847	24.26 days	<0.001	HR = 0.727 (0.663.0.798)	Adjusted HR = 0.689 (0.627.0.757)
	Aspirin users	764/3,847	-			
**90-day mortality**	Non-users	1,388/3,847	24.26 days (21.42, 27.10)	<0.001	HR = 0.759 (0.700.0.822)	Adjusted HR = 0.722 (0.666, 0.783)
	Aspirin users	1,055/3,847	46.47 days (38.63, 54.31)			

### 4.3 Association of aspirin with composite outcomes

The aspirin group had a shorter average duration of ICU and no significant difference in gastrointestinal hemorrhage rate was found between the two groups (Table 3).

**TABLE 3:** Composite outcomes of the aspirin use group and the non-users group.

Composite outcomes	Non-users	Aspirin users	*p*-value
Length of ICU stay, median (inter-quartile range)	5.58 (3.27.10.17)	5.19 (3.17.9.57)	0.006
Gastrointestinal hemorrhage; n (%)	40 (1%)	28 (0.7%)	0.144

### 4.4 Subgroup analyses

**FIGURE 4:**
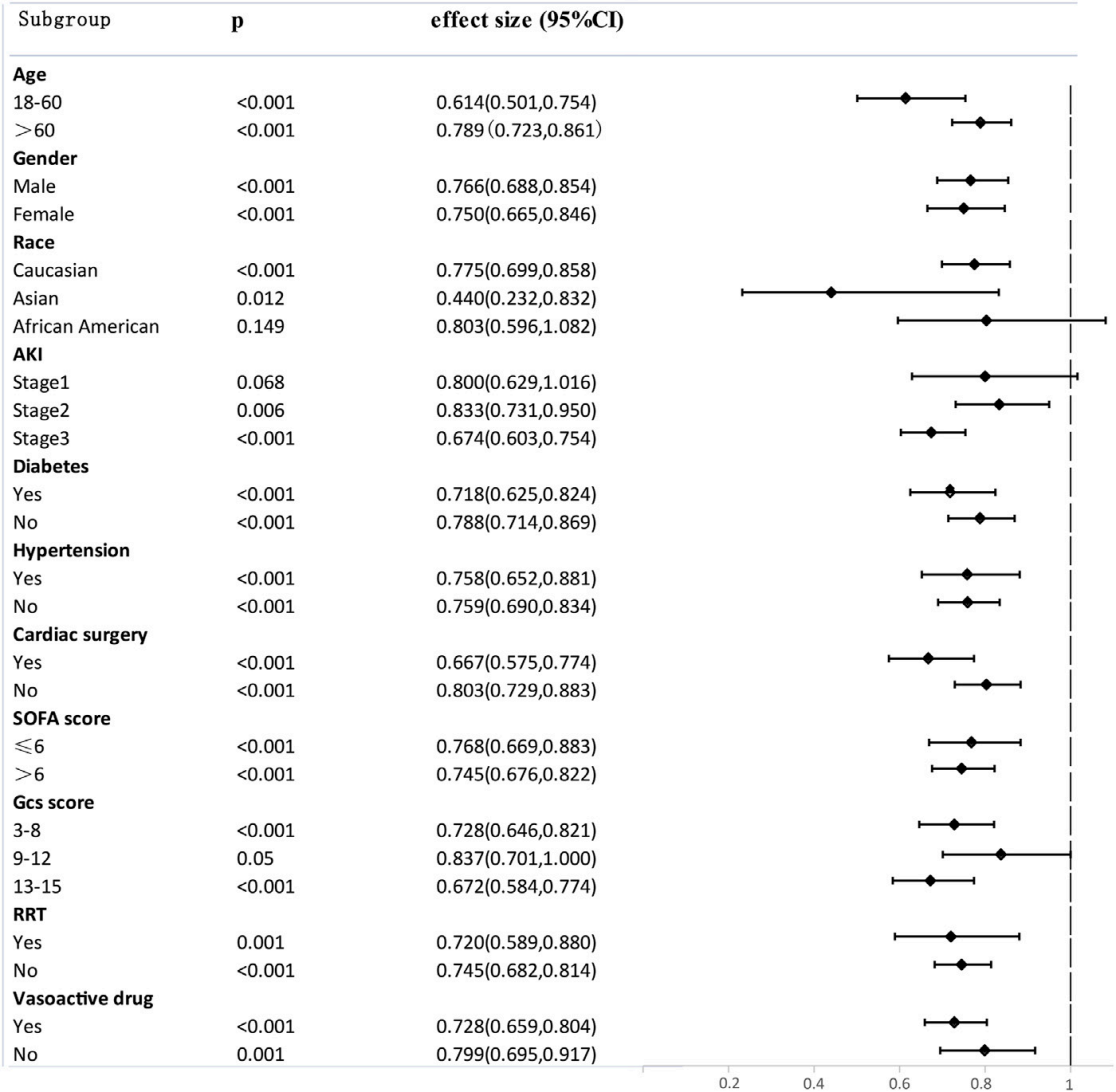
Subgroup analysis of the relationship between aspirin and 90-Day mortality, illustrated by a Forest Plot.

SA-AKI patients were divided into different subgroups according to age, gender, race, AKI stage, co-morbidity, SOFA scores, GCS score, renal replacement therapy (RRT), and vasoactive drug. The effect of aspirin on 90-day mortality in the SA-AKI subgroups was investigated, and the results were illustrated as a Forest Plot (Figure 4). The results revealed that aspirin was associated with lower 90-day mortality in the following subgroups: age ≤60 years (HR 0.614, 95% CI 0.501.0.754), Asian (HR 0.440, 95% CI 0.232.0.832), SOFA score > 6 points (HR 0.745, 95% CI 0.676.0.822), GCS score ≥ 13 points (HR 0.672, 95% CI 0.584.0.774), Aki stage 3 (HR 0.674, 95% CI 0.603.0.754), cardiac surgery (HR 0.667, 95% CI 0.575.0.774), diabetes (HR 0.718, 95% CI 0.625.0.824), using Vasoactive drug (HR 0.728, 95% CI 0.659.0.804), and using RRT (HR 0.720, 95% CI 0.589.0.880). There is dramatic difference of Hazard Ratio (HR) between difference race. We found that compared with Asian (HR 0.440, p 0.012, 95% CI 0.232.0.832) and Caucasian (HR 0.775, *p* < 0.001, 95% CI 0.699.0.858), the benefit of using aspirin in African American SA-AKI patients is limited (HR 0.803, p 0.149, 95% CI 0.596.1.082). That may explain the conflicting opinions of previous studies about the impact of aspirin on SA-AKI patients. Our subgroup analyses may contribute to personalized treatment of aspirin.

### 4.5 Dose of aspirin

Prostaglandins cyclooxygenase has two isoforms: COX-1 and COX-2 (Mitchell et al., 1993). Low-doses aspirin is a more potent inhibitor of COX-1 and thus platelet activation (COX-1-mediated) can be inhibited, while high-dose aspirin inhibits both COX-1- and COX-2-dependent prostanoid generation (Ornelas et al., 2017; Mirabito Colafella et al., 2020). In order to further explore the optimal dose of aspirin therapy, we analyzed the relationship between the dose of aspirin and the 90-day mortality by Cox proportional-hazards model.

**FIGURE 5:**
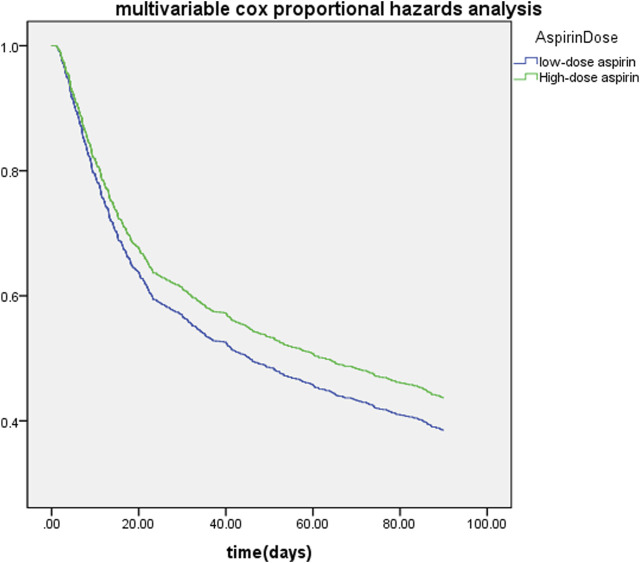
Cox proportional-hazards model of the dose of aspirin and 90-day mortality in SA-AKI patients. Low-dose (300 mg/d) aspirin group is represented by blue lines and high-dose (>300 mg/d) aspirin group is represented by green lines.

SA-AKI patients which have used aspirin in hospitalization were divided into different groups according to the dose of aspirin. Compared to the low-dose (≤ 300 mg/d) aspirin, Lower 90-day mortality risks were observed in high-dose (>300 mg/d) aspirin group (adjusted HR 0.852, 95% CI 0.744, 0.977, *p* = 0.02) (Table 4) and Cox proportional-hazards model was displayed in Figure 5.

**TABLE 4:** Survival outcomes of the high-dose aspirin group and the low-dose aspirin group.

	Group	No.events/No. All patients	Median survival time (95%CI)	Log rank test value	Hazard ratio (95%CI)	Adjusted hazard Ratio (95%CI)
**90-day mortality**	low-dose aspirin group	753/2,781	42.07 days (34.07, 50.06)	0.043	HR = 0.871 (0.762.0.995)	Adjusted HR = 0.852 (0.744, 0.977)
	high-dose aspirin group	302/1,066	60.34 days (-, -)			

In general, we conclude that for SA-AKI patients, high-dose (>300 mg/d) aspirin may be preferable to conventional low-dose (≤ 300 mg/d) aspirin. Therefore, our research indicated that aspirin might reduce the mortality risks in SA-AKI patients by influencing both COX-1 and COX-2.

## 5 Discussion

The benefits of using aspirin in SA-AKI patients remain unknown, and the current observational study assess whether using aspirin is beneficial to the prognosis of SA-AKI patients. This study demonstrated that treatment with aspirin in SA-AKI patients admitted to the ICU might reduce the 90-day and 30-day mortality risks. Furthermore, no significant difference in gastrointestinal hemorrhage was found between the aspirin group and the non-aspirin group.

Despite advances in sepsis and SA-AKI research, the mortality remains high. Sepsis is described as an uncontrolled inflammatory and procoagulant response to pathogens, and platelets play a key role in inducing microvascular thrombosis and releasing inflammatory mediators by platelet–endothelial interaction (Semple et al., 2011). Sepsis activates platelets, resulting in systemic thrombosis and driving the multi-organ failure of DIC (Cox, 2023). Infection activates the innate immune system response mediated by platelets (Engelmann and Massberg, 2013) and if this response persists, it will enhance thrombus formation (Stark and Massberg, 2021). During sepsis, platelet activation causes damage to endothelial cells and promotes neutrophil extracellular trap and microthrombus formation, exacerbating septic coagulation and inflammatory reactions and aggravating organ damage (Wang et al., 2018). Inhibition of platelet function may be an effective way to attenuate inflammatory reactions and coagulation in sepsis models, as well as reduce damage to organ function (Akinosoglou and Alexopoulos, 2014).

Platelet activation also have been reported plays a significant part in AKI because it disturbs renal haemodynamic processes which leads to sustained hypoxaemic renal tissue injury (Jansen et al., 2018). And platelet can activate other platelets and endothelial cells through expressing CD40, release cytokines and chemokines, and activate complement to facilitate inflammation during AKI (Jansen et al., 2018). An observational study of 770 receiving cardiac surgery patients revealed that continuous low-dose (75mg/100 mg) aspirin was protective against postoperative AKI (OR 0.39, 95% CI 0.22-0.67, *p* = 0.001) (Hur et al., 2017). Aspirin was the first antiplatelet agent administered in the clinical setting and is still widely used today. By inhibiting COX-1, it prevents the synthesis of thromboxane A2, one of powerful platelet activators (Sagar et al., 2020).

The benefit of aspirin in sepsis and SA-AKI patients is most likely related to its anti-platelet properties. Multiple relevant studies have shown that antiplatelet medications decrease mortality in sepsis and SA-AKI patients. According to a 10-year large cohort research, people receiving antiplatelet medications at the time of sepsis diagnosis may have a decreased risk of mortality (aOR 0.78, 95% CI 0.76–0.79) (Tsai et al., 2015). A secondary analysis from a prospective, multicenter assessment of severe sepsis in Japan revealed that the in-hospital mortality rate of patients receiving antiplatelet drugs as pretreatment was considerably lower than patients who did not receive antiplatelet therapy (OR 0.51, 95% CI: 0.32–0.82, *p* = 0.006) (Kobayashi et al., 2022). A meta-analysis, including 10 cohort studies and 689,897 patients with sepsis, suggested that hospital or ICU mortality of sepsis patients was significantly decreased by aspirin (OR = 0.60, 95% CI: 0.53-0.68, *p* < 0.05) (Ouyang et al., 2019). Moreover, in the subgroup analysis of this study, anti-platelet therapy after sepsis was associated with a lower mortality risk (OR = 0.59, 95% CI: 0.52-0.67) than initiating anti-platelet therapy before sepsis (OR = 0.78, 95% CI: 0.77-0.80). Therefore, anti-platelet function of aspirin may be one of reason for increasing 90-day survival rate in sepsis and SA-AKI.

The three fundamental mechanisms of SA-AKI include microvascular dysfunction, inflammation, and metabolic reprogramming (Peerapornratana et al., 2019). Aspirin has powerful anti-inflammatory properties, which are attributed to inhibiting prostanoid biosynthesis and generating lipoxins and Resolvin (Spite and Serhan, 2010). Previous research reported that aspirin-triggered resolvin D1 (AT-RvD1) effectively downregulated inflammatory responses in lipopolysaccharide (LPS)-induced AKI mice thereby diminishing renal tubular damage in endotoxin-induced acute kidney injury (Chen et al., 2014). Resolvin D1 (RvD1) is an anti-inflammatory bioactive compound that can downregulate NF-κB inflammatory signals and inhibit renal cell apoptosis, highlighting its potential as a therapeutic target in septic AKI (Zhao et al., 2016). 17(R)-hydroxy docosahexaenoic acid is derived from aspirin-acetylated cyclooxygenase-2 (COX-2) and can be converted to 17 R-RvD (Serhan et al., 2002).

The dosage of aspirin determines its impact (Patrono and Baigent, 2019). Low-dose aspirin can irreversibly inhibit COX-1 and thus reduce the ability of platelets production and secrete the secondary platelet agonist thromboxane A2, while high-dose aspirin inhibits both COX-1 and COX-2 thereby activating the ability of anti-inflammation (Ornelas et al., 2017). Previous research compared the effects of low-dose (COX-1 inhibition) and high-dose (both COX-1 and COX-2 inhibition) aspirin on kidney damage during angiogenesis-inhibitor therapy in rodents and found that only high-dose aspirin could prevent albuminuria (Mirabito Colafella et al., 2022). In our study, compared to the low-dose (≤ 300 mg/d) aspirin, lower 90-day mortality risks were observed in high-dose (>300 mg/d) aspirin group (adjusted HR 0.852, 95% CI 0.744, 0.977, *p* = 0.02) (Table4). Therefore, we supposed that both anti-platelet and anti-inflammatory functions of aspirin may benefit SA-AKI patients.

The other reason for that high-dose (>300 mg/d) works better may be the type of aspirin. Previous study has reported that equivalent doses of the enteric-coated aspirin were not as effective as plain aspirin due to its lower bioavailability and poor absorption from the small intestine with higher pH environment (Cox et al., 2006) so 75 mg enteric-coated aspirin daily may be incomplete in many patients (Maree et al., 2005). Researchers found that a considerable part of non-responders show adequate COX inhibition by using equivalent doses plain of aspirin to replace the enteric-coated (Peace et al., 2010). Some on enteric-coated low dose aspirin are not receive the full anti-platelet effects may be one of reasons to explain lower 90-day mortality risks were observed in high-dose (>300 mg/d) aspirin group.

Aspirin is usually prescribed as secondary prevention to improve cardiovascular disease survival outcomes by antiplatelet aggregation and to prevent atherothrombosis. It can selectively acetylate Ser-529 close to the catalytic pocket of the enzyme, thus irreversibly inactivating platelet cyclooxygenase (COX)-1 and suppressing TXA2 generation (Patrono et al., 2011). The results of subgroup analyses in our study showed that cardiac surgery (HR 0.667, 95% CI 0.575.0.774) patients, compared with non-cardiac surgery patients (HR 0.803, 95% CI 0.729.0.833), probably benefit more from using aspirin as secondary prevention (Figure 4). Hypertension patients (HR 0.758, 95% CI 0.652.0.881) and diabetes patients (HR 0.718, 95% CI 0.625.0.824) also benefit more from using aspirin compared with non-hypertension patients (HR 0.759, 95% CI 0.690.0.834) and non-diabetes patients (HR 0.788, 95% CI 0.714.0.869) (Figure 4). A significant proportion of SA-AKI patients also have a history of hypertension, diabetes mellitus, and cardiac surgery which are hazardous factors for cardiovascular-related mortality. Aspirin might protect against SA-AKI by exerting beneficial effects on the cardiovascular system.

Nevertheless, the limitations of the current study should be acknowledged. Firstly, we just analyzed the most severe critically ill patients since MIMIC database consists of ICU admissions in the Beth Israel Deaconess Medical Center. Although propensity score matching was used to narrow the gap of baseline characteristics between the two groups, the clinical conditions of ICU are complicated and the mortality of these patients may be caused by other factors not aspirin. Secondly, due to the observational study design, the group assignment was not randomized. Although PSM was used to minimize bias between the aspirin group and the non-aspirin group, residual confounding factors may have influenced the prognosis. Thirdly, this is a retrospective study, and the causality of the correlation between aspirin use and SA-AKI outcomes cannot be confirmed. Fourth, true baseline SCr measurements which may influence AKI grading were not available for all patients. Finally, the effects of aspirin on long-term kidney outcomes could not be evaluated due to the lack of long-term data. Therefore, animal experiment and additional prospective cohort studies should be conducted to explore the association between aspirin and prognosis in SA-AKI patients.

## 6 Conclusion

Aspirin might reduce the average duration of ICU and 30-day or 90-day mortality risks in SA-AKI patients. No significant difference in gastrointestinal hemorrhage rates was found between the aspirin group and the non-aspirin group. Aspirin may be an effective drug for SA-AKI. However, further studies are required to investigate the specific mechanism underlying aspirin benefiting SA-AKI patients.

## Data Availability

Publicly available datasets were analyzed in this study. This data can be found here: https://physionet.org/content/mimiciv/2.1/(certification number: 42257067).
