# Supplementary material for: Aspirin reduces the mortality risk of sepsis-associated acute kidney injury: an observational study using the MIMIC IV database
Source: Front Pharmacol. 2023 Jul 25;14:1186384. doi: 10.3389/fphar.2023.1186384 (PMC10407089; doi:10.3389/fphar.2023.1186384)
Supplement: Supplementary file 2 [file Table1.DOCX]

**Table S1.The number (%) of missing variables in our research**

| **variables** | **Missing number (%)** |
| --- | --- |
| age | 0 |
| gender | 0 |
| race | 0 |
| heart_rate_mean | 20 (0.17%) |
| sbp_mean | 65 (0.54%) |
| dbp_mean | 66 (0.55%) |
| spo2_mean | 20 (0.17%) |
| diabete | 0 |
| hypertension | 0 |
| cardiac_surgery | 0 |
| RRT | 0 |
| mechanical_ventilation | 0 |
| vasoactive drug | 0 |
| urineoutput | 238 (1.94%) |
| hemoglobin_min | 15 (0.12%) |
| wbc_min | 14 (0.12%) |
| platelets_min | 14 (0.12%) |
| glucose_min | 55 (0.45%) |
| pt_min | 520 (4.30%) |
| gcs_min | 3 (0.02%) |
| Sofa | 0 |
| los_icu | 0 |
| admittime | 0 |
| Aspirin | 0 |
| gastrointestinal hemorrhage | 0 |

Abbreviations: sbp: systolic blood pressures; dbp: diastolic blood pressures; spo2:oxygen saturation; RRT: renal replacement therapy; wbc: white blood cells; los_icu: length of stay at intensive care unit;pt: prothrombin time; gcs: Glasgow Coma Scale; sofa: Glasgow Coma Scale
